# Applications of Unmanned Aerial Vehicle Based Imagery in Turfgrass Field Trials

**DOI:** 10.3389/fpls.2019.00279

**Published:** 2019-03-15

**Authors:** Jing Zhang, Simerjeet Virk, Wesley Porter, Kevin Kenworthy, Dana Sullivan, Brian Schwartz

**Affiliations:** ^1^Crop and Soil Sciences Department, University of Georgia, Tifton, GA, United States; ^2^Agronomy Department, University of Florida, Gainesville, FL, United States; ^3^Turf Scout LLC., Greensboro, NC, United States

**Keywords:** bermudagrass, zoysiagrass, drought, normalized difference vegetation index, visible atmospherically resistant index, remote sensing

## Abstract

Recent advances in remote sensing technology, especially in the area of Unmanned Aerial Vehicles (UAV) and Unmanned Aerial Systems (UASs) provide opportunities for turfgrass breeders to collect more comprehensive data during early stages of selection as well as in advanced trials. The goal of this study was to assess the use of UAV-based aerial imagery on replicated turfgrass field trials. Both visual (RGB) images and multispectral images were acquired with a small UAV platform on field trials of bermudagrass (*Cynodon* spp.) and zoysiagrass (*Zoysia* spp.) with plot sizes of 1.8 by 1.8 m and 0.9 by 0.9 m, respectively. Color indices and vegetation indices were calculated from the data extracted from UAV-based RGB images and multispectral images, respectively. Ground truth measurements including visual turfgrass quality, percent green cover, and normalized difference vegetation index (NDVI) were taken immediately following each UAV flight. Results from the study showed that ground-based NDVI can be predicted using UAV-based NDVI (*R*^2^ = 0.90, RMSE = 0.03). Ground percent green cover can be predicted using both UAV-based NDVI (*R*^2^ = 0.86, RMSE = 8.29) and visible atmospherically resistant index (VARI, *R*^2^ = 0.87, RMSE = 7.77), warranting the use of the more affordable RGB camera to estimate ground percent green cover. Out of the top ten entries identified using ground measurements, 92% (12 out of 13 in bermudagrass) and 80% (9 out of 11 in zoysiagrass) overlapped with those using UAV-based imagery. These results suggest that UAV-based high-resolution imagery is a reliable and powerful tool for assessing turfgrass performance during variety trials.

## Introduction

Constraints such as high demands of time and labor during field phenotyping limit the ability of turfgrass breeders to collect more comprehensive data during early stages of selection and later in advanced trials. Recent improvements in remote sensing technologies provide opportunities to mitigate this bottleneck for future breeding advances. Non-invasive remote sensing methods such as digital image analysis and spectral reflectance have been widely used for quantifying turfgrass cover and quality (Menges et al., 1985; Richardson et al., 2001; Jiang and Carrow, 2007; Xiong et al., 2007). Vegetation indices such as NDVI and ratio vegetation index (RVI), calculated from reflectance at red and near infrared (NIR) bands, have been validated to predict turfgrass health in previous studies (Fitz-Rodríguez and Choi, 2002; Jiang and Carrow, 2007; Bremer et al., 2011). UAV platforms are evolving rapidly and offer advantages over other vehicles when used for remote sensing. When compared with satellite-based and ground-based remote sensing, UAV-based imagery has higher spatial resolution (1–2 cm per pixel) (Xiang and Tian, 2011) and thus more powerful statistical analytics at an affordable price. Both spatial and temporal resolution are important for turfgrasses because they can experience intermittent drought stresses in sandy soils in only three or 4 days without precipitation (Zhang et al., 2018).

In agricultural studies, the use of UAV-based imagery is increasing. High-resolution digital images have been acquired from aerobatic model aircrafts for estimating the nutrient status and crop biomass of corn (*Zea mays* L.), alfalfa (*Medicago sativa* L.), and soybeans [*Glycine max* (L.) Merr.] (Hunt et al., 2005). Primicerio et al. (2012) developed a six-rotor aerial platform with a multi-spectral camera to map the vineyard vigor of wine grapes (*Vitis vinifera* L.). In more recent studies, UAV systems were applied to monitor crop growth in wheat (*Triticum* spp.) (Lelong et al., 2008; Torres-Sánchez et al., 2014), predict yield in rice (*Oryza Sativa* L.) (Zhou et al., 2017), detect disease in potato (*Solanum tuberosum* L.) (Sugiura et al., 2016), and detect weeds in sunflower (*Helianthus annuus* L.) fields (Torres-Sánchez et al., 2013).

Recent studies with UAVs on turfgrass have also shown promising results. Xiang and Tian (2011) used an unmanned helicopter to monitor turfgrass response after glyphosate application and found only a 1.5% difference in the estimation of herbicide damage between aerial images and ground surveys. Caturegli et al. (2016) used UAV-based multi-spectral imagery to estimate the nitrogen status of hybrid bermudagrass (*C. dactylon* L. × *C. transvaalensis* Burtt-Davy), zoysiagrass (*Z. matrella* L. Merr.), and seashore paspalum (*Paspalum vaginatunt* Swartz.), concluding that UAV imagery can adequately assess the spatial variability of nitrogen status for these turfgrass species in large areas such as golf courses and sod farms. Another study assessed the health of creeping bentgrass (*Agrostis stolonifera* L.) using a RGB camera and a modified NIR camera under different mowing heights (Sommer et al., 2017). It was reported that color indices calculated from RGB images were better correlated (*R*^2^∼0.8) with ground truth data compared to mNDVI from the modified NIR camera (*R*^2^∼0.6). To our knowledge, no investigation has been conducted regarding the use of UAV-based imagery on turfgrass variety trials. Prior to being used by turfgrass breeders in variety trials, data extracted based on UAV images need to be compared with ground measurements. Therefore, the overall goal of the present study was to assess the potential use of visual (RGB) and multispectral images collected with a UAV platform in replicated turfgrass field trials. The objectives were (1) to examine the correlation between UAV-based measurements and ground measurements; (2) to determine the feasibility of developing a general model to predict ground measurements for two turfgrass species in different sampling dates; and (3) to assess if the information extracted from UAV-based imagery can help turfgrass breeders to make better decisions (genotype rankings) during selection processes.

## Materials and Methods

### UAV System and Cameras

**FIGURE 1:**
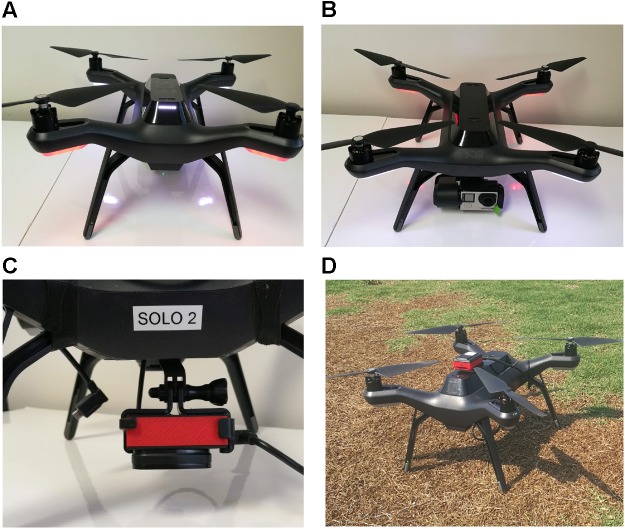
**(A)** Back view of Unmanned aerial vehicle (3DR Solo quadcopter). **(B)** Front view of UAV with GoPro RGB camera. **(C)** Parrot Sequoia multispectral camera mounted on UAV. **(D)** UAV with multispectral camera waiting to take off in the field.

A Solo quadcopter (3D Robotics, Berkeley, CA, United States) with vertical take-off and landing capabilities was used to collect aerial images for this study (Figure 1). The UAV system, equipped with four brushless motors, can fly by either remote control or autonomously with Global Positioning Systems (GPS) and waypoint navigation system. The whole system consists of the drone, controller, and a ground station with software for mission planning, flight control and telemetry (Torres-Sánchez et al., 2013). The ground station interface between the pilot-in-command and the UAV has supporting software that implements a flight plan and monitors the flight. The software also has a telemetry system that collects relevant flight data and information including GPS position data and flight time. This information is known as the telemetry log and is useful during image processing. Two persons were involved during the flight mission, one being the remote pilot who was in charge of taking-off and landing the UAV by activating the programmed flight plan during the flight operations, and the second who acted as a visual observer that kept watch of the UAV for potential collision threats with other air traffic. Two cameras mounted separately on two similar quadcopters were used in this study. The first was a GoPro Hero 4 (GoPro, Inc. San Mateo, CA, United States) visual camera which acquires 7-megapixel images in true color (Red, R; Green, G, and Blue, B, bands) with 8-bit radiometric resolution (Figure 1B). The second camera was a Parrot Sequoia (MicaSense, Seattle, WA, United States) multispectral camera that measures at four narrow spectral bands (green: 530–570 nm; red: 640–680 nm; red edge: 730–740 nm; NIR: 770–810 nm) (Figure 1C,D).

### Study Site and UAV Flights

The study site was part of a multi-state breeding project funded by U.S. Department of Agriculture – Specialty Crop Research Initiative. Two advanced breeding trials of bermudagrass and zoysiagrass were planted using plugs in June 2016 on a loamy sand (Tifton-Urban land complex, pH 5.3) at the University of Georgia Tifton Campus. Field plots were arranged as a randomized complete block design with three replications. The plot size was 1.8 by 1.8 m for bermudagrass and 0.9 m by 0.9 m for zoysiagrass. Forty advanced lines from two breeding programs (University of Georgia and Oklahoma State University) and four commercial cultivars (“Celebration,” “Latitude 36,” “TifTuf,” and “Tifway”) were included in the bermudagrass trial. In the zoysiagrass study, 40 advanced lines from two breeding programs (Texas A&M and University of Florida) and 3 commercial cultivars (“Empire,” “Palisades,” and “Zeon”) were tested. The UAV-based RGB and multispectral images were taken on 28 September 2017, 5 April 2018, 8 May 2018, and 18 June 2018 at 0930 h with no cloud cover. The flight altitude was 30 m for the RGB camera, resulting in image resolution of 2.2 cm per pixel. For the multispectral camera, the flight altitude was 46 m (to aid in image stitching later because the multispectral camera has a narrower field of view) which resulted in an image resolution of 4.3 cm per pixel. The UAV speed was set to 4.5 ms^-1^ for all flights. All flights were controlled using a free software, “Tower Beta” (DroidPlanner, 2018), installed on a smart device (Tablet) and by creating a flight plan for UAV equipped with each camera prior to any flight. The software maintained the desired flight altitude and flight speed during the flight while capturing the images at 80% front and side overlaps.

### Image Preprocess and Data Acquisition

**FIGURE 2:**
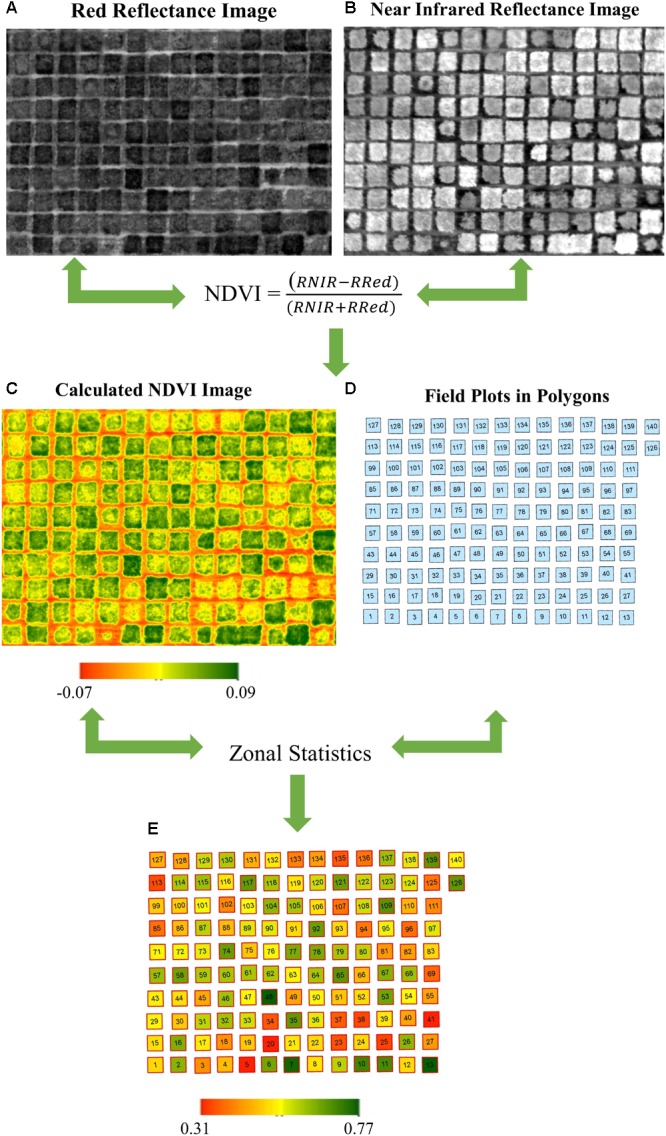
Workflow of raster image process and data acquisition. **(A)** Reflectance image on red band. **(B)** Reflectance image on near infrared band. **(C)** Calculated NDVI image using a. and b. through the equation. **(D)** Polygons represent individual field plot. **(E)** Average NDVI value for each plot using zonal statistics based on **C** and **D**. For illustration purpose, the plots are bermudagrass plots.

Geotagging of the RGB images collected by the GoPro Hero 4 was completed using the information provided by the telemetry log saved on the smart device and using the open source software, ‘Mission Planner’ (Oborne, 2018). Multispectral images were geotagged during flight with an on-board GPS in the Parrot Sequoia camera. Geotagged images were then processed and stitched in Pix4Dmapper Pro 4.2.27 (Pix4D SA, Lausanne, Switzerland) to generate an orthomosaic consisting of information collected for individual bands (RGB, NIR, and Red). Standard templates of “Ag RGB” and “Ag Multispectral” in Pix4Dmapper were used for stitching RGB and multispectral images respectively. The georeferenced orthomosaic was exported in a TIFF format for further analysis in ArcGIS (Esri, Redlands, CA, United States). For data analysis, a shape file consisting of the individual field plot information was created in ArcMap version 10.4.1 (Esri, Redlands, CA, United States). Data were extracted within each polygon (each polygon represented a plot) using the ArcMap feature in zonal statistics (Figure 2). Three different vegetation indices including NDVI, Green NDVI (GNDVI), and Normalized Difference Red Edge Index (NDRI) were calculated from the multispectral images (Table 1). Six color indices were calculated with normalized values for R, G, and B bands from the digital image (Torres-Sánchez et al., 2014; Saberioon et al., 2014; Zhou et al., 2017). The following normalization scheme was applied to the color indices:

**Table 1:** Equations for color indices used in the study.

Indices	Equation	Reference
NDVI: normalized difference vegetation index	(R_NIR_−R_Red_)/(R_NIR_+R_Red_)	Lee et al., 2011
GNDVI: green normalized difference vegetation index	(R_NIR_−R_Green_)/(R_NIR_+R_Green_)	Gitelson et al., 1996
NDRI: normalized red edge index	(R_NIR_−R_Rededge_)/(R_NIR_+R_Rededge_)	Mutanga and Skidmore, 2004
NDI: normalized difference index	(g−r)/(g+r)	Woebbecke et al., 1995
ExG: excessive green index	2g−r−b	Woebbecke et al., 1995
ExR: excessive red	1.4r−g	Meyer and Neto, 2008
ExGR: excessive green index minus excess red index	3g−2.4r−b	Meyer and Neto, 2008
VARI: visible atmospherically resistant index	(g−r)/(g+r−b)	Gitelson et al., 2002
GLI: green leaf index	(2g−b−r)/(2g+b+r)	Louhaichi et al., 2001

r=R/(R+G+B); g=G/(R+G+B); b=B/(R+G+B)

where R, G, and B are the values of the red, green, and blue bands, respectively. Those six color indices included normalized difference index (NDI), excessive green index (ExG), excessive red (ExR), excessive green index minus excess red index (ExGR), VARI, and green leaf index (GLI) (Table 1).

### Ground Measurements

Ground truth measurements including turfgrass quality (TQ), percent green cover, and canopy spectral reflectance of the plots were collected on the same day within an hour or two of the UAV-based imagery data collection. Visual ratings of TQ were based on the National Turfgrass Evaluation Program using a 1 to 9 scale (9 = excellent performance and 1 = poor performance, 6 = minimum acceptable quality) (Morris and Shearman, 2008). Percent green cover was estimated from digital images collected using a digital camera (Powershot G5; Canon, Tokyo, Japan) mounted to an enclosed photo box (56 cm by 56 cm) with four 9-W compact fluorescent lamps (TCP; Lighthouse Supply, Bristol, VA, United States). Each image was analyzed using SigmaScan Pro (version 5.0; Systat Software, San Jose, CA, United States) for percent green cover (0–100%) using a hue range from 60 to 120 and saturation range from 10 to 100 as outlined by Richardson et al. (2001). Canopy spectral reflectance values were measured using a Crop Circle ACS470 sensor (Holland Scientific, Lincoln, NE, United States), equipped with a decimeter level GPS (Raven Industries, Sioux Falls, SD, United States). The spectral sensor, with active light source, measured light reflectance in three spectral bands centered on 550 nm (green), 650 nm (red), and 730 nm (NIR). The system was mounted to a mobile cart at 61 cm above ground with a target ground area of 35 by 6.4 cm. Data were collected and processed using TurfScout platform (TurfScout, Greensboro, NC), where NDVI values were calculated within the program.

### Data Analysis

All data were subjected to analysis of variance using SAS 9.4 (SAS Institute Inc., Cary, NC, United States). For each parameter, Fisher’s protected LSD at 0.05 probability level was used to separate significant means and to mark the top statistical group in bermudagrass and zoysiagrass entries. Turf Performance Index was calculated by summing up the number of times an entry entered the top statistical group (Wherley et al., 2013). Spearman’s rank correlation and linear regression were performed between UAV-based measurements and ground measurements in SAS using the CORR and Generalized Linear Model (GLM) procedures, respectively. Graphs were generated using SigmaPlot 14 (Systat Software, Inc. Point Richmond, CA, United States). Box plots were generated using ggplot2 in Rstudio (Wickham, 2016).

## Results and Discussion

### Distribution of Ground Truth Measurements and UAV-Based Measurements

**FIGURE 3:**
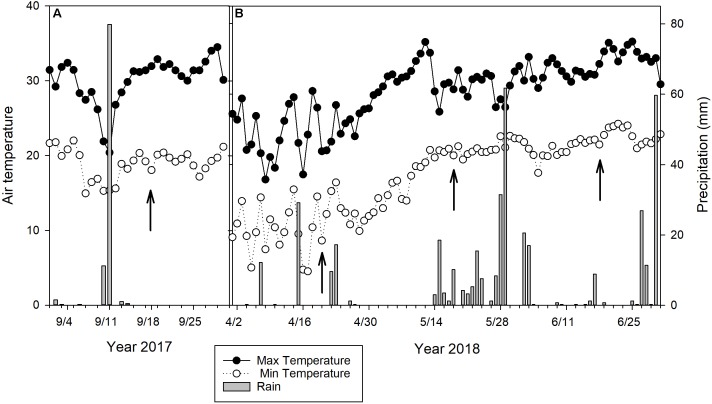
Maximum and minimum daily temperature and precipitation of the study area in **(A)** September 2017 and **(B)** April, May, and June 2018. Arrows indicate when the data were collected.

**FIGURE 4:**
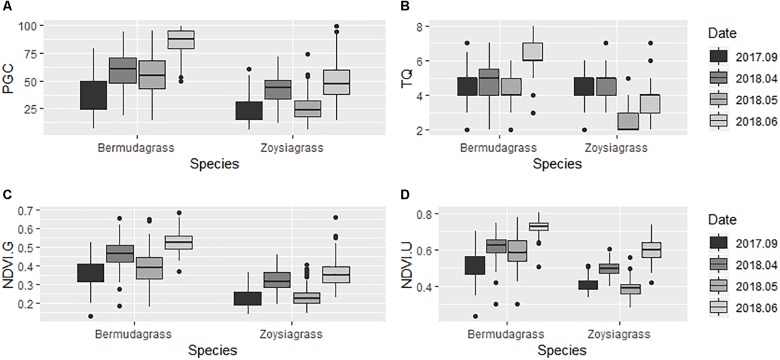
Boxplots of **(A)** Percent green cover (PGC). **(B)** Turfgrass quality (TQ). **(C)** Ground NDVI. **(D)** UAV-based NDVI in bermudagrass and zoysiagrass field trials from September 2017 to June 2018.

Ground percent green cover, TQ, ground and UAV-based NDVI in bermudagrass ranged from 10 to 100%, 2 to 8, 0.17 to 0.66, and 0.35 to 0.80, respectively (Figure 2). The aforementioned parameters in zoysiagrass ranged from 10 to 80%, 2 to 6, 0.10 to 0.53, and 0.28 to 0.74, respectively. Parameters such as percent green cover and NDVI were affected by different sampling dates due to the combination of temperature, irradiance, and precipitation. In general, sampling dates in September 2017 and April 2018 were drier than the other two dates. Weather data were obtained from a weather station located about 1 kilometer away from the study site (Figure 3). Although no soil moisture data and leaf water content was taken, virtual symptoms of leaf wilting under varying degrees was observed in the plots due to the combination of evapotranspiration demand and irrigation frequency (once every 2 weeks). The study site was originally established to evaluate long-term drought resistance in advance experimental lines, and the irrigation frequency was designed to encourage periodic drought. Zoysiagrasses responded more rapidly compared to bermudagrasses in these drier environments as indicated by percent green cover and NDVI (Figure 4). This was likely due to the relative poor drought resistance of zoysiagrass as compared to that of bermudagrass (Zhang et al., 2018). Seasonal effects and interaction between turfgrass species and environments are not the focus of this study. Data over different seasons and species were collected to better elucidate the relationship between ground truth and UAV-based measurements and to build a more robust model.

### Correlation Between Ground Measurements and UAV-Based Measurements

For multispectral imagery, ground truth measurements including TQ, percent green cover, and ground NDVI were positively correlated with the UAV-based vegetation indices NDVI (0.64, 0.89, and 0.92, *P* < 0.0001), GNDVI (0.59, 0.82, and 0.88, *P* < 0.0001), and NDRI (0.60, 0.68, and 0.76, *P* < 0.0001), respectively (Table 2). For RGB imagery, the aforementioned ground truth measurements were positively correlated with the UAV-based color indices VARI (0.59, 0.88, and 0.88, *P* < 0.0001), NDI (0.48, 0.56, and 0.60, *P* < 0.0001), ExG (0.44, 0.66, and 0.67, *P* < 0.0001), ExR (−0.28, −0.70, and −0.68, *P* > 0.0001), ExGR (0.53, 0.79, and 0.79, *P* < 0.0001), and GLI (0.63, 0.79, and 0.81, *P* < 0.0001), respectively. Further results and discussion is focused on UAV-based NDVI and VARI due to their higher correlation with the ground truth measurements in the study.

**Table 2:** Spearman’s rank correlation coefficients between ground measurements (TQ, PGC, and NDVI.G) and UAV-based measurements (NDVI.U, GNDVI.U, NDRI.U, VARI, NDI, ExG, ExR, ExGR, and GLI) in bermudagrass and zoysiagrass variety trials from September 2017 to June 2018 in Tifton, GA.

	Ground measurements			UAV-based Measurements							
	PGC	NDVI.G	NDVI.U	GNDVI.U	NDRI.U	VARI	NDI	ExG	ExR	ExGR	GLI
TQ†	0.65	0.66	0.64	0.59	0.60	0.59	0.48	0.44	−0.28	0.53	0.63
PGC		0.88	0.89	0.82	0.68	0.88	0.56	0.66	−0.70	0.79	0.79
NDVI.G			0.92	0.88	0.76	0.88	0.60	0.67	−0.68	0.79	0.81
NDVI.U				0.98	0.76	0.91	0.49	0.80	−0.81	0.90	0.90
GNDVI.U					0.73	0.85	0.38	0.82	−0.82	0.90	0.89
NDRI.U						0.70	0.41	0.67	−0.59	0.72	0.76
VARI							0.69	0.69	−0.78	−0.78	0.84
NDI								0.12	−0.19	0.30	0.38
ExG									−0.89	0.95	0.93
ExR										−0.92	−0.82
ExGR											0.97

*TQ, turfgrass quality; PGC, percent green cover; NDVI.G, normalized difference vegetation index at ground level; NDVI.U, UAV-based NDVI; RNDVI.U, UAV-based green NDVI; NDRI.U, UAV-based normalized difference red edge index; VARI, UAV-based visible atmospherically resistant index; NDI, normalized difference index; ExG, excess green; ExR, excess red; ExGR, excess green minus excess red; GLI, green leaf index. †All coefficients are significantly at 0.001 P level except when labeled with “ns.”*

### Predicting Ground Measurements Using UAV-Based Measurements

**FIGURE 5:**
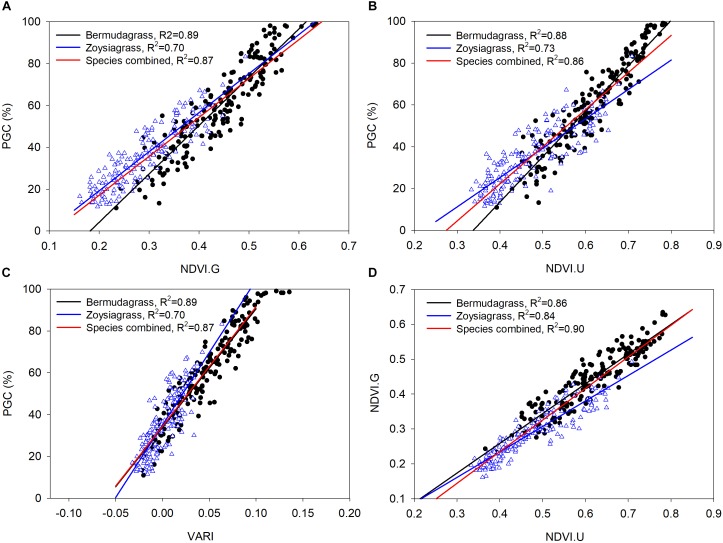
Linear regressions in predicting percent green cover (PGC) using **(A)** Ground NDVI, **(B)** UAV-based NDVI, and **(C)** VARI. **(D)** In predicting ground NDVI using UAV-based NDVI. Triangle points represented zoysiagrass and circled points represented bermudagrass.

Regression analysis indicated that ground percent green cover in bermudagrass can be predicted using UAV-based NDVI (PGC = 217.11 × NDVI. U – 73.27, *R*^2^ = 0.88, RMSE = 7.37) and VARI (PGC = 567.35 × VARI + 33.86, *R*^2^ = 0.89, RMSE = 6.94) (Table 3 and Figure 5) with only ∼7% difference according to the root mean square errors. Similarly, models fitted to predict ground percent green cover in zoysiagrass had R^2^ of 0.73 (PGC = 140.39 × NDVI.U – 30.88, RMSE = 7.70) and 0.69 (PGC = 693.31 × VARI + 35.02, RMSE = 8.24) using UAV-based NDVI and VARI, respectively. Ballesteros et al. (2014) also found that the color index VARI was a good predictor for estimating percent green cover in maize (PGC = 293.8 × VARI + 67.13, *R*^2^ = 0.95, RMSE = 6.71) and onion (*Allium cepa* L.) (PGC = 279.1 × VARI + 68.13, *R*^2^ = 0.82, RMSE = 6.29). Models of row crops like maize and onion had steeper slopes in predicting percent green cover than turfgrass in our study, which can be caused by multiple factors. Firstly, maize and onion crops have wider leaves than turfgrass so percent green cover maybe more responsive to the change in VARI. Secondly, for row crops, percent green cover is more of an interest before canopy closure and this would likely to impact the threshold settings in obtaining percent green cover. In turfgrass, percent green cover is used throughout every stage of growth. Therefore, results on row crops may not be directly applicable for turfgrass. When data from both the bermudagrass and zoysiagrass experiments were combined, ground NDVI was still accurately predicted using UAV-based NDVI (*R*^2^ = 0.84–0.86, RMSE = 0.03), indicating that UAV-based sensor information is consistent across at least these two turfgrass species. Sommer et al. (2017) reported that color indices (*R*^2^ = 0.80) from a RGB camera were better correlated with ground measurement NDVI than mNDVI (*R*^2^ = 0.60) from a modified NIR camera. In our study, VARI was a good predictor for ground percent green cover in our study, supporting the use of a more affordable digital camera for data collection if ground percent green cover is the interest. Multi-spectral cameras may provide more information for the detection of weed pressure (Torres-Sánchez et al., 2013), disease incidence (Mahlein, 2016; Brodbeck et al., 2017), and drought stress (Zarco-Tejada et al., 2013).

**Table 3:** Regressions between ground measurements including percent green cover (PGC), turfgrass quality (TQ), and ground normalized difference vegetation index (NDVI.G) and UAV-based measurements including NDVI.G and visible atmospherically resistant index (VARI).

	Adjusted *R*^2^	*p*-value	RMSE†
**Bermudagrass**			
PGC (%) = 217.11 × NDVI.U − 73.27	0.88	^∗∗∗^	7.37
PGC (%) = 567.35 × VARI + 33.86	0.89	^∗∗∗^	6.94
TQ = 9.19 × NDVI.U − 0.58	0.68	^∗∗∗^	0.58
TQ = 22.92 × VARI + 4.00	0.63	^∗∗∗^	0.62
NDVI.G = 0.85 × NDVI.U − 0.08	0.86	^∗∗∗^	0.03
**Zoysiagrass**			
PGC (%) = 140.39 × NDVI.U − 30.88	0.73	^∗∗∗^	7.70
PGC (%) = 693.31 × VARI + 35.02	0.69	^∗∗∗^	8.24
TQ = 4.41 × NDVI.U + 1.78	0.13	^∗∗∗^	1.04
TQ = 14.17 × VARI +3.86	0.05	^∗∗^	1.09
NDVI.G = 0.73 × NDVI.U − 0.06	0.84	^∗∗∗^	0.03
**Species Combined**			
PGC (%) = 177.49 × NDVI.U − 48.74	0.86	^∗∗∗^	8.29
PGC (%) = 571.61 × VARI + 34.38	0.87	^∗∗∗^	7.77
TQ = 7.47 × NDVI.U + 0.41	0.49	^∗∗∗^	0.87
TQ = 23.21 × VARI + 3.92	0.47	^∗∗∗^	0.89
NDVI.G = 0.91 × NDVI.U − 0.13	0.90	^∗∗∗^	0.03

*^∗∗^ and ^∗∗∗^ indicate model significance level at 0.01, 0.001 P level. †RMSE is the standard deviation of the error.*

All models for the sensor-based prediction of TQ were more poorly fitted (*R*^2^ = 0.63–0.68 for bermudagrass and 0.05–0.13 for zoysiagrass) than those for percent green cover. This agrees with previous findings that TQ is more difficult to predict based on percent green cover or NDVI (Sullivan et al., 2017), which was largely due to its subjective nature and greater coefficients of variation than optical sensor measurements (Bell et al., 2002; Lee et al., 2011; Bremer et al., 2011). Although TQ, based on 1–9 scale, is still an important parameter to be used when evaluating turf performance, objective measurements such as percent green cover and NDVI are being adopted more often for their reliable and repeatable estimation of quality (Richardson et al., 2001, 2008, Bremer et al., 2011). Results from our study demonstrated great potential for UAV-based imagery to be used in assessing turf performance.

Bremer et al. (2011) reported that separate models for different grass species may be required to predict TQ with ground NDVI. To date we have not found a report on whether ground-based NDVI or percent green cover of turfgrass can be predicted using UAV-based measurements, or whether these relationships are species dependent. In our study, the model based on combined data from both species had an increased *R*^2^ (0.90) and equal root mean square of error (RMSE = 0.03) in the prediction of ground NDVI using UAV-based NDVI compared to models fitted from individual species (Table 3). Using a species combined model to predict percent green cover in bermudagrass resulted in an increased error of 0.92% in UAV-based NDVI and 0.83% in VARI. The increase in errors were smaller when predicting zoysiagrass percent green cover (0.59% for NDVI.U and 0.47% for VARI). These results suggest that developing a general model to predict ground NDVI using a UAV platform for both bermudagrass and zoysiagrass, and possibly more species, without a significant sacrifice of accuracy is possible. However, before building a general model to increase the efficiency of data collection, certain issues need to be addressed and the limitation of the current model should be taken into account. Several UAV-based platforms and sensors need to be tested for the same purpose in order to determine if the model is platform/sensor dependent. The model may be limited when it comes to a different turfgrass species with broader leaf such as St. Augustinegrass [*Stenotaphrum secundatum* (Walt.) Kuntze] and under less ideal weather conditions, such as overcast and cloudy days. Compared to different species and weather conditions, repeated time scale is less of a concern for fitting the model. Further investigation, addressing those limitations, should be completed to improve the fit of general models to predict ground measurements from UAV-based systems.

### Entry Ranking Using Ground Measurements and UAV-Based Measurements

Depending on the trait or genes targeted for improvement, typically the top performing 10–20% (Hanna et al., 2013) of genotypes are advanced to the next cycle of selection during early generation turfgrass breeding variety trials, which in the context of this study represents the best 5–10 performers. There was a 100% overlap in the identification of the top 5 bermudagrass entries using ground measurements (percent green cover and NDVI) and UAV-based measurements (NDVI and VARI) (Table 4), including Tif16118 (1st vs. 1st), Tif16116 (1st vs. 5th), Tif16115 (3rd vs. 2nd), TifTuf (3rd vs. 3rd), Tif16110 (3rd vs. 5th), and Tif16112 (3rd vs. 5th). An overlap of 93% was observed in identifying the top 10 bermudagrass entries between two groups of measurements, meaning 12 out of 13 identified by ground measurements were the same entries identified using UAV-based measurements. Less overlap (55%) was found in identifying the top 5 zoysiagrass entries using both types of measurements, but 80% of the top 10 zoysiagrass entries corresponded to ground and UAV-based rankings. The 55% overlap of the top 5 zoysiagrasses was due to the lack of statistical separation among 5th ranking entries using ground measurements, including DALZ1614, Empire, FZ1440, FAES1335, FAES1322, DALZ1625, and DALZ1410.

**Table 4:** Turf performance index (TPI) and genotypic ranking using ground measurements (percent green cover and ground NDVI) and UAV-based measurements (NDVI and VARI) in bermudagrass and zoysiagrass from September 2017 to June 2018.

	Ground		UAV			Ground		UAV	
Bermudagrass	TPI†	Rank	TPI‡	Rank	Zoysiagrass	TPI	Rank	TPI	Rank
Tif16118	8	1	8	1	DALZ1409	7	1	8	1
Tif16116	8	1	5	5	DALZ1604	7	1	8	1
Tif16115	7	3	7	2	DALZ1606	7	1	5	5
TifTuf	7	3	6	3	Palisades	7	1	4	10
Tif16110	7	3	5	5	DALZ1614	5	5	7	3
Tif16112	7	3	5	5	Empire	5	5	6	4
OSU1337	6	7	6	3	FZ1440	5	5	5	5
Tif16106	6	7	3	10	FAES1335	5	5	4	10
OSU1439	6	7	3	10	FAES1322	5	5	4	10
OSU1406	5	10	5	5	DALZ1625	5	5	3	20
Tif16102	5	10	4	9	DALZ1410	5	5	2	32
Tif16104	5	10	3	10	FZ1427	4	12	5	5
Tif16117	5	10	0	27	DALZ1605	4	12	4	10
OSU1408	4	14	3	10	FZ1436	4	12	3	20
Tif16105	4	14	3	10	FZ1327	4	12	2	32
Tif16114	4	14	1	18	DALZ1611	3	16	4	10
Tif16108	3	16	3	10	DALZ1311	3	16	3	20
OSU1433	3	16	1	18	DALZ1603	3	16	3	20
OSU1418	3	16	1	18	FAES1313	3	16	3	20
Tif16103	3	16	0	27	DALZ1609	3	16	3	20
Tif16107	3	16	0	27	FZ1309	3	16	3	20
Tif16101	3	16	0	27	DALZ1408	3	16	2	32
Tif16113	2	23	2	16	DALZ1313	3	16	2	32
Tifway	2	23	2	16	FZ1252	3	16	2	32
OSU1412	2	23	1	18	FZ1410	3	16	2	32
Latitude36	2	23	1	18	FZ1407	2	26	4	10
OSU1402	2	23	1	18	FAES1336	2	26	4	10
OSU1403	2	23	1	18	FZ1429	2	26	4	10
OSU1417	2	23	0	27	DALZ1310	2	26	3	20
OSU1257	2	23	0	27	Zeon	2	26	2	32
Tif16119	2	23	0	27	DALZ1626	2	26	1	40
OSU1435	2	23	0	27	FZ1337	2	26	1	40
OSU1414	1	33	1	18	FZ1422	1	33	5	5
OSU1425	1	33	0	27	DALZ1613	1	33	4	10
Tif16109	1	33	0	27	FAES1337	1	33	3	20
Tif16120	1	33	0	27	DALZ1601	1	33	3	20
OSU1423	1	33	0	27	DALZ1607	1	33	3	20
Tif16111	1	33	0	27	FZ1333	1	33	1	40
OSU1318	0	38	1	18	DALZ1314	0	39	5	5
Celebration	0	38	0	27	DALZ1615	0	39	4	10
OSU1310	0	38	0	27	FAES1307	0	39	3	20
OSU1409	0	38	0	27	FAES1319	0	39	2	32
OSU1415	0	38	0	27	FAES1312	0	39	1	40
OSU1420	0	38	0	27					

*†Ground TPI summed up the number of times an entry entered the top statistical group in percent green cover and ground NDVI. ‡UAV TPI summed up the number of times an entry entered the top statistical group in UAV-based NDVI and VARI.*

Stress tolerance is a common interest in turfgrass variety trials (Chandra, 2015), with performance during drought stress being of the utmost importance. In our study, both ground and UAV-based measurements were used to rank the entries from all dates combined and also only during the two drier dates (September 2017 and May 2018). There was an 83% overlap in the identification of the top 5 entries in both species over all dates, and when the two drier dates were analyzed separately (Table 5). A few entries had an increased ranking under conditions of reduced soil moisture, indicating some mechanism of drought tolerance or avoidance. These entries were Tif16115 (from 2nd to 1st) and TifTuf (from 2nd to 1st) bermudagrass, DALZ1614 (from 4th to 2nd), FA1427 (from 7th to 5th), and FAES1322 (from 9th to 5th) zoysiagrass.

**Table 5:** Turf performance index (TPI) based on TQ, percent green cover, ground NDVI, UAV-based NDVI and VARI and genotypic ranking based on TPI in bermudagrass and zoysiagrass from September 2017 to June 2018.

	All dates		Drought			All dates		Drought	
Bermudagrass	TPI†	Rank	TPI‡	Rank	Zoysiagrass	TPI	Rank	TPI	Rank
Tif16118	19	1	10	1	DALZ1409	19	1	10	1
Tif16115	17	2	10	1	DALZ1604	19	1	9	2
TifTuf	17	2	10	1	DALZ1606	16	3	8	5
OSU1337	16	4	6	9	DALZ1614	15	4	9	2
Tif16110	16	4	8	5	Empire	14	5	9	2
Tif16116	16	4	9	4	Palisades	14	5	7	8
Tif16112	15	7	6	9	FAES1335	13	7	7	8
OSU1406	14	8	7	6	FZ1427	13	7	8	5
Tif16106	12	9	7	6	DALZ1605	12	9	6	10
OSU1439	11	10	3	15	FAES1322	12	9	8	5
Tif16102	11	10	7	6	FZ1440	12	9	6	10
Tif16104	11	10	5	12	DALZ1311	10	12	4	19
OSU1408	9	13	4	13	DALZ1611	10	12	6	10
Tif16105	9	13	6	9	DALZ1625	10	12	5	13
OSU1412	6	15	3	15	FZ1422	10	12	4	19
OSU1433	6	15	3	15	DALZ1410	9	16	5	13
Tif16108	6	15	1	21	FAES1313	9	16	5	13
Tif16113	6	15	1	21	FAES1336	9	16	5	13
Tif16114	6	15	1	21	FZ1436	9	16	3	30
Tif16117	6	15	4	13	DALZ1314	8	20	5	13
Tifway	5	21	2	18	DALZ1408	8	20	4	19
OSU1417	4	22	2	18	DALZ1603	8	20	3	30
OSU1418	4	22	1	21	DALZ1609	8	20	4	19
Tif16103	4	22	1	21	FZ1327	8	20	4	19
Tif16107	4	22	0	33	FZ1407	8	20	4	19
Latitude36	3	26	1	21	FZ1429	8	20	4	19
OSU1257	3	26	1	21	DALZ1310	7	27	3	30
OSU1402	3	26	1	21	DALZ1313	7	27	4	19
OSU1403	3	26	1	21	DALZ1613	7	27	4	19
Tif16101	3	26	1	21	FAES1337	7	27	2	37
Tif16119	3	26	2	18	FZ1252	7	27	4	19
OSU1414	2	32	0	33	FZ1309	7	27	5	13
OSU1425	2	32	1	21	Zeon	6	33	4	19
OSU1435	2	32	1	21	DALZ1615	5	34	3	30
Tif16109	2	32	0	33	FAES1307	5	34	3	30
Tif16120	2	32	0	33	FAES1319	5	34	3	30
OSU1318	1	37	0	33	FZ1410	5	34	2	37
OSU1423	1	37	0	33	DALZ1601	4	38	2	37
Tif16111	1	37	0	33	DALZ1607	4	38	3	30
Celebration	0	40	0	33	DALZ1626	4	38	2	37
OSU1310	0	40	0	33	FZ1337	4	38	2	37
OSU1409	0	40	0	33	FAES1312	3	42	1	42
OSU1415	0	40	0	33	FZ1333	3	42	1	42
OSU1420	0	40	0	33					

*†All dates TPI summed up the number of times an entry entered the top statistical group for all five parameters on all four dates. ‡Drought dates TPI summed up the number of times an entry entered the top statistical group for all five parameters on September 2017 and May 2018.*

In summary, predicting objective variables such as ground percent green cover and NDVI is more reliable than subjective visual TQ. Data (VARI and NDVI) extracted based on high-resolution UAV imagery provide accurate estimates of ground measurement such as percent green cover and NDVI. Given the advantages in field of view, spatial and temporal resolution, UAV-based imagery would equip turfgrass breeder with powerful tools for assessing turfgrass performance, which would greatly increases the efficiency of data collection in relatively large trials (>1,000 plots). Before developing a general model for predicting ground measurement for turfgrass, further investigation of more UAV platforms/sensors on different turfgrass species under varying weather conditions and locations is needed. Further research using more advanced thermal and hyperspectral sensors should be conducted during periods of drought stress, as well as for disease detection, to determine their value against more readily available UAV-based imagery platforms.

## Author Contributions

JZ and BS designed the experiments. JZ, SV, and WP collected UAV images. JZ and BS took the ground measurements. JZ processed the images and analyzed the data. DS contributed the data analysis on the ground measurement of NDVI. KK provided critical reviews and supported the costs of the study.

## Conflict of Interest Statement

The authors declare that the research was conducted in the absence of any commercial or financial relationships that could be construed as a potential conflict of interest.
